# Chronic Suppuration in the Nasal Accessory Sinuses and Its Treatment

**Published:** 1906-08-25

**Authors:** Wm. J. Chichele Nourse

**Affiliations:** Surgeon to the Central London Throat, Nose, and Ear Hospital; Late President of the British Laryngological, Rhinological, and Otological Association.


### Hospital Clinics.

### CHRONIC SUPPURATION IN THE NASAL ACCESSORY SINUSES AND ITS TREATMENT.

Two Lectures by Wm. J. Chichele Nourse, F.R.C.S.(Edin.), Surgeon to the Central London Throat, Nose, and Ear Hospital; late President of the British Laryngological, Rhinological, and Otological Association.

(*Continued from page* 346.)

### II. THE TREATMENT.

#### Puncture of the Maxillary Antrum.

Puncture of the maxillary antrum through the inferior meatus of the nose, besides being necessary for diagnosis, also constitutes the first step in the treatment. At this stage we are often not yet able to decide whether the pus in the sinus is due to sinusitis or simply to empyema.

A 10-per-cent. solution of cocain, with a little adrenalin, is applied to the inferior meatus on a small piece of absorbent cotton, so as to shrink the parts and render them insensitive. After a few minutes a long fine trocar (Lichtwitz) is passed under the inferior turbinal body about an inch behind its anterior end until it touches the outer wall of the nose rather above the level of the floor, and is pushed outwards, backwards, and slightly upwards into the antrum. After withdrawing the stylet the sinus should be thoroughly washed out through the cannula and perflated with a strong current of air, and the middle meatus is to be dried with small pieces of wool and examined for signs of pus coming from elsewhere. Any carious stumps in the upper jaw on the affected side should be extracted.

The next time the patient is seen the nasal puncture should be repeated, or the sinus may be washed out through the natural ostium by means of Killian's cannula. Boric lotion, being clear and unirritating, is the best to use at first.

The plan, formerly so much in vogue, of puncturing the antrum through a tooth-socket after the extraction of a tooth has nothing to recommend it, and, moreover, has distinct drawbacks. It has been altogether given up by the author.

Puncture and irrigation of the antrum through the inferior meatus with Lichtwitz' trocar is almost painless, and can be repeated as often as is necessary. If the case is one of empyema from a dental source, and the diseased tooth has been removed, a very few such washings will usually be sufficient for a cure. If it is empyema of the nasal type, even though the source has not been dealt with, a few irrigations will much diminish the quantity of pus found in the sinus, and render it inodorous. In true chronic sinusitis the quantity of pus washed out each time remains about the same, and the fœtor persists.

#### Perforation Through the Inferior Meatus.

Now and then cases occur in which, although the disease is rebellious to simple treatment, it is not advisable for some reason to perform a radical operation, or the patient declines it. In such cases a permanent opening may be made into the antrum through the inferior meatus by using a larger trocar (Krause's), followed by a burr, cutting forceps, or an angular knife, the anterior extremity of the inferior turbinal having been previously removed if it is in the way. For this operation a short general anæsthesia is required. The patient can then be taught how to introduce a curved metal cannula into the antrum from the nose and to wash out the cavity once or twice daily.

#### The Radical Operation.

In true chronic maxillary sinusitis a radical operation is the only certain method of cure. The principle of the Caldwell-Luc operation, which presents the radical surgical treatment in its most approved form, consists in making use of a large opening in the anterior wall of the sinus for inspecting and treating its interior, after which a large permanent opening is made from the antrum into the nose by removing a large part of the nasal wall, and finally the buccal wound is closed. The results of this operation are excellent.

#### Claoue's Modification.

Claoué has proposed to do without the opening in the anterior wall, and to curette the sinus through the nasal opening. His operation may be occasionally useful, but it lacks one great advantage offered by the other—that the field of operation is open to the view, so that recesses and incomplete septa can be recognised and dealt with. To ensure a good result every shred of the diseased lining-membrane must be removed. Chloride of zinc solution should then be applied and the cavity packed with a long strip of ribbon-gauze, of which one end hangs in the nostril. The gauze is left in place for twenty-four hours.

#### Treatment of Chronic Frontal Sinusitis.

The treatment of chronic frontal sinusitis should be commenced by the removal of the anterior extremity of the middle turbinal body and of any polypi in that region, and by irrigations through the cannula, followed by perflation of air, repeated at intervals.

For injections in bulk, warm lotions of boric acid or some other mild antiseptic may be used. One part of hydrogen peroxide solution diluted with two parts of water and a very dilute solution of lysoform are useful. Besides these, small quantities of stronger remedies may be injected, the head being bent forward directly afterwards, while the cavity is perflated, so that the fluid can run out without coming in contact with other sensitive regions in the nose. A number of different astringents and stimulants, including oily solutions containing menthol, have been tried by the author, with variable results. Liquids may also be driven into the sinus through the cannula in the form of nebulæ

by means of compressed air, and antiseptic vapours such as that of Kelvolin.

If the fronto-nasal canal is small, and the drainage does not seem to be free, it is a good plan to pass a drainage-tube into the sinus from the nose. It is perfectly possible to do so by using the frontal probe as a guide, and having a second piece of tube on the probe behind the first for pushing it into its place. The effect of leaving a small tube in the fronto-nasal canal for a few days is to dilate it, so that it will admit one of larger calibre. A drainage-tube may be allowed to remain in the fronto-nasal canal for a fortnight without risk. Care should be taken, however, that it is of a proper length. If it is too long the free end is apt to cause discomfort by flapping to and fro in the nostril; if too short, it may slip entirely into the sinus. A suitable length is about 6.5 cm. The author's conclusion is that it is possible, by means of intra-nasal treatment, to reduce the discharge almost to a minimum and to get rid of the symptoms, at any rate temporarily, but that these measures cannot be relied on to produce a cure. In all uncomplicated cases intra-nasal treatment should first be tried for some time. It is probable that few cases of chronic frontal sinusitis are really curable except by operation; but in deciding whether it is necessary in a particular case, many factors must be taken into account, including the degree to which the symptoms can be controlled by milder treatment.

### The Ogston-Luc Operation Preferred.

The Ogston-Luc operation is that generally preferred; in favourable cases the results are most satisfactory, but, as pointed out by Luc himself at the Congress of the American Laryngological Society in 1903, there are other cases in which it does not give satisfactory results.

The size of the frontal sinus is very variable; it may be a small pyramidal cavity, or it may have a limb extending outwards towards the zygoma, another upwards, and a third backwards over the roof of the orbit. This is one cause of the unsatisfactory cases; another is the participation of ethmoidal cells in the suppurative process, which leads to a re-infection of the frontal sinus while there are still granulations in it, and the healing process is incomplete. For these cases Killian's operation is the one to be selected. In the Ogston-Luc operation an incision is made through the inner part of the eyebrow, running on to the root of the nose. The periosteum is retracted and an opening made in the bone with a small trephine or a sharp gouge and mallet in the anterior wall of the sinus just above the inner angle of the orbit. After the interior of the sinus has been explored with a probe and its extent determined, more of the anterior wall can be removed so as to allow of free access to all parts of the cavity. It is not necessary to take away all of the anterior wall. The whole of the diseased lining is then removed with curettes and forceps, and an antiseptic applied.

The anterior extremity of the middle turbinal has been removed beforehand, but it is necessary to make sure that there is a free opening from the sinus into the nose.

### Drainage.

Some surgeons then pass a strip of gauze from the sinus into the nose, but the author prefers the original plan of using a rubber drainage-tube, which, however, should be of such a size as to completely fill the fronto-nasal canal.

A long silver probe, bent into a curve nearly that of three-quarters of the circumference of a circle 7 cm. in diameter, with a large eye, will pass easily from the frontal wound out of the nostril, carrying a stout silk thread. The thread is then secured to a piece of tubing, which is drawn down until the upper end only just projects into the sinus. The lower end is cut off about a centimetre beyond the nasal orifice. If there is a difficulty in getting the tube into place its calibre can be temporarily reduced by stretching it; when released, its elasticity causes it to fill the canal completely. The frontal wound is then closed and a dressing applied.

The healing sinus is thus kept completely shut off from the nose and from any septic foci in the ethmoidal cells. The drainage-tube is left in until it becomes loose after several days and slips out. The sinus is not irrigated. The lower end of the tube is kept clean by swabs moistened with biniodide solution, and, if necessary, the nose is syringed occasionally with Dobell's solution, avoiding the tube.

### Kilian's Operation.

Killian's operation is more complicated. The whole of the anterior wall of the sinus is removed, as well as the floor, but leaving the bony margin of the orbit with the periosteum covering it. The frontal process of the superior maxilla is also taken away, so as to obtain access to the ethmoidal cells, which are removed as far as the disease extends.

In cases of combined suppuration, when the frontal and maxillary sinuses both contain pus, it is best, probably, to operate on the frontal sinus first, having previously punctured the antrum and washed out its contents and removed the anterior end of the middle turbinal body. When the frontal sinus has healed, the condition of the antrum can be tested again. If it still contains pus a radical operation is required.

### Suppuration in the Ethmoidal Cells.

The treatment of suppuration in the ethmoidal cells is begun by removing the polypi with which the nostril is often filled. The snare is the best instrument to use. If they are numerous the process may require several sittings, as it is not prudent to do too much at once, especially in elderly patients. The anterior end of the middle turbinal, or, if necessary, the whole of it, should then be removed, and the suppurating cells opened up until free drainage has been secured, and all loculi of pus destroyed. For these proceedings punch-forceps of one or two patterns, and Moure's duckbill forceps, or Luc's broad nasal forceps, are required. For the posterior ethmoidal cells, Hajek's hooks and a cutting instrument by Cordes are of great use.

### Sphenoidal Sinus.

When the sphenoidal sinus is affected it should be washed out a few times with an antiseptic lotion, such as well-diluted lysoform, when this is possible; but one of the difficulties about this sinus is the small size of its ostium, which may only admit a fine probe, or it may just be possible to insert a cannula, which fits the orifice so tightly as not to allow the exit of air or liquid. In these conditions, or when the symptoms continue in spite of irrigation, portions of the middle turbinal must be removed until the ostium of the sinus comes into view, preparatory to making a breach in its anterior wall. The instruments preferred by the author are a graduated steel probe with a flattened end and Cordes' instrument. The latter is particularly useful for enlarging ostia of very small size. When the front blade has passed through the ostium small pieces of the bony margin can be nipped off and the opening enlarged in any direction.

As much as possible of the anterior wall of the sinus should be taken away, so as to ensure a permanent opening and free drainage. The sinus is then treated with an antiseptic and plugged with a tampon soaked in carbolised glycerin, attached to a silk thread, which is left hanging outside the nostril. The plug should be renewed daily. In all except the worst cases this treatment is sufficient, provided the opening is made large enough. This point is important, as there is a remarkable tendency for the opening to close.

In conclusion, it may be added that there are numerous points of interest concerning diagnosis and treatment which have been left altogether untouched in this short *resumé*.

